# Heterotaxy in *Caenorhabditis*: widespread natural variation in left–right arrangement of the major organs

**DOI:** 10.1098/rstb.2015.0404

**Published:** 2016-12-19

**Authors:** Melissa R. Alcorn, Davon C. Callander, Agustín López-Santos, Yamila N. Torres Cleuren, Bilge Birsoy, Pradeep M. Joshi, Anna W. Santure, Joel H. Rothman

**Affiliations:** 1 Department of MCD Biology and Neuroscience Research Institute, University of California, Santa Barbara, CA 93106, USA; 2 School of Biological Sciences, University of Auckland, Auckland, New Zealand; 3 Department of MCD Biology, University of Colorado, Boulder, CO 80309-0347, USA

**Keywords:** GWAS, heterotaxy, *C. elegans*, left–right asymmetry, natural variation

## Abstract

Although the arrangement of internal organs in most metazoans is profoundly left–right (L/R) asymmetric with a predominant handedness, rare individuals show full (mirror-symmetric) or partial (heterotaxy) reversals. While the nematode *Caenorhabditis elegans* is known for its highly determinate development, including stereotyped L/R organ handedness, we found that L/R asymmetry of the major organs, the gut and gonad, varies among natural isolates of the species in both males and hermaphrodites. In hermaphrodites, heterotaxy can involve one or both bilaterally asymmetric gonad arms. Male heterotaxy is probably not attributable to relaxed selection in this hermaphroditic species, as it is also seen in gonochoristic *Caenorhabditis* species. Heterotaxy increases in many isolates at elevated temperature, with one showing a pregastrulation temperature-sensitive period, suggesting a very early embryonic or germline effect on this much later developmental outcome. A genome-wide association study of 100 isolates showed that male heterotaxy is associated with three genomic regions. Analysis of recombinant inbred lines suggests that a small number of loci are responsible for the observed variation. These findings reveal that heterotaxy is a widely varying quantitative trait in an animal with an otherwise highly stereotyped anatomy, demonstrating unexpected plasticity in an L/R arrangement of the major organs even in a simple animal.

This article is part of the themed issue ‘Provocative questions in left–right asymmetry’.

## 1. Introduction

The arrangement of internal organs in most metazoans is substantially left–right (L/R) asymmetric with a largely invariant handedness. Molecular and cellular mechanisms that break L/R symmetry and subsequently determine internal organ placement have been illuminated in a variety of animal systems [1–7]. While these mechanisms are largely conserved within a phylum, it is clear that the control systems that create and implement L/R asymmetry and handedness are widely divergent between phyla [8,9]. Although the innovation of handedness-determining systems appears to have occurred frequently during metazoan evolution, the evolutionary basis for establishing new mechanisms of handedness asymmetry is generally unknown. In the context of L/R axis formation, two major questions persist: how is a particular body plan selected for on evolutionary timescales [10]? And how is this L/R positional information established and maintained during development [11–18]?

The asymmetry of the L/R axis is established during early embryogenesis and persists throughout development, as reflected in the overall body plan. However, subsequent symmetry breaks that are largely independent of earlier L/R asymmetries give rise to additional L/R differences, including in specific structures (e.g. handedness of heart looping [19,20], laterality in the central nervous system [21–23] and motor handedness [24–27]). In humans, complete L/R reversal of internal organ structure (*situs inversus totalis*) occurs in rare individuals, generally with no known ill consequences. However, disruption of internal organ asymmetries can result in birth defects with varying severity, most notably in cases in which two or more organs deviate from the canonical structure or position [28], a condition known as heterotaxy, which is frequently associated with ciliopathies [29,30].

The multiplicity of L/R symmetry breaks that generate anatomical, neuronal and behavioural bilateral asymmetries occurs even in simple animals, including the nematode *Caenorhabditis elegans* [18,31–34], which contains only approximately 1000 somatic cells. The major structural chirality of the *C. elegans* embryo is established during the division of the two anterior granddaughters of the zygote [35]. Anteroposterior skewing of mitotic spindles with a defined handedness, driven by actomyosin-directed chiral cortical flows that generate torque as these cells divide [36], creates an embryo with defined (‘dextral’) chirality. As a result, progeny cells subsequently make bilaterally asymmetric contacts with Notch-signalling cells, resulting in differential cell fate specification on opposite sides on the embryo [37]. Many later L/R asymmetries, including differences in cell identities, specificity of bilateral pairs of neurons [38] and the stereotyped L/R arrangement of the gut and gonad [35], are prefigured by this early symmetry-breaking event. However, L/R differences between some cell types that are made stochastically [39], and the L/R motor handedness bias of male mating [24], are independent of this embryonic chirality. Further, we found that the stereotyped L/R arrangement of the intestine and gonad of males in the laboratory isolate is frequently reversed at elevated temperature, independent of the overall anatomical handedness of the animal (electronic supplementary material, table S1 and figure S2; [40]), suggesting that the symmetry-breaking mechanism leading to the bilateral orientation of these organs is at least partially separable from the event that creates chirality of the embryo.

Identifying mechanisms that engender L/R asymmetries independent of embryonic chirality, and an understanding of the evolutionary basis for changes in these events, can be illuminated by analysing potential incipient changes in the processes between evolutionarily divergent isolates of a species with variation in L/R asymmetries. We report here substantial variation in the arrangement of the major organs (the gut and gonad) in both sexes among a set of naturally inbred *C. elegans* wild isolates representing 97 distinct haplotypes, as well as in other *Caenorhabditis* species, including both hermaphroditic and gonochoristic species. Although all descendants from each of these naturally inbred *Caenorhabditis* isolates are isogenic, we found that heterotaxy occurs between genetically identical individuals of many isolates. Genome-wide association studies reveal that this frequent heterotaxy is associated with three chromosomal regions, demonstrating that heterotaxy is a genetically heritable trait and a substrate for natural selection. These findings reveal that heterotaxy of the two major organs is a common trait in an animal with an otherwise highly determinate anatomy, and that the L/R arrangement of these organs is subject to substantial variation during the radiation of a single species.

## 2. Results

### (a) The fidelity of left–right gut/gonad asymmetry varies widely across wild isolates of *Caenorhabditis elegans* males

The chirality of *C. elegans* embryos is established during the four- to six-cell stage of embryogenesis, as a result of skewing of the mitotic spindles in descendants of the anterior daughter of the zygote, AB [35]. This symmetry break, which occurs with a virtually invariant L/R handedness (‘dextral’ chirality), has been shown to lead to nearly all of the stereotyped L/R asymmetries in cell type and organ arrangement in the mature animal [41]. This bilateral asymmetry includes the reproducible L/R orientation of the major internal organs, the gut and gonad, which is readily scored at low magnification in a dissecting microscope and has been used as a proxy for overall L/R anatomical handedness [17]. Healthy animals of either sex with sinistral anatomical handedness can be generated as a result of physical manipulation of the mitotic spindles, perturbation of the integrity of the eggshell or exposure to low temperature [42]. In addition, viable sinistral animals can arise as a result of mutations in *gpa-16* [17], which encodes a small rho-GTPase required for normal spindle placement, and in the actin cytoskeleton [32], leading to reversal of AB spindle skewing. However, several studies of a large number (more than 15 000) of animals [17,35,40,42] reared at 20°C indicated that such L/R left–right reversals in the arrangement of the major organs in the laboratory N2 isolate do not occur under normal conditions, suggesting that evolutionary constraints have led *C. elegans* to adopt a virtually invariant dextral body plan.

**Figure 1.**
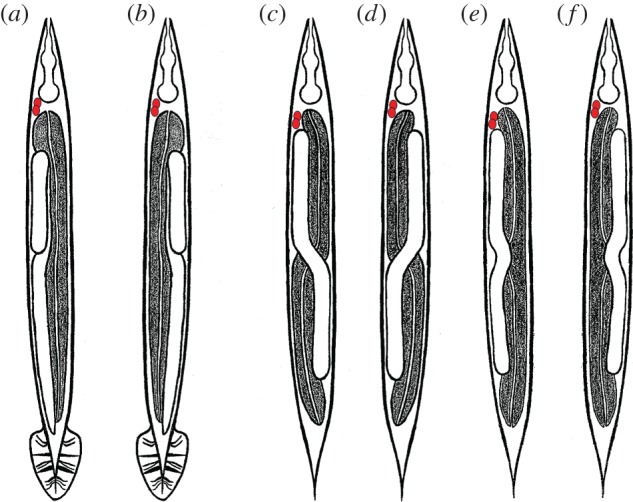
Schematic of gut/gonad heterotaxy in *Caenorhabditis* males and hermaphrodites. The anterior pair of coelomocytes, ccAR and ccPR (marked in red), is found on the right side of normal (dextral) worms and is used as reference for the L/R handedeness. The black texture marks the gut, while the gonad is in white. All animals are shown in a ventral view with anterior up. (*a*) Dextral (normal) male: the coelomocytes and gonad are positioned on the right side of the animal, with the gut on the left, (*b*) reversed male: the position of the gut and gonad are reversed with the coelomocytes at their normal right-side position, (*c*) dextral hermaphrodite: the anterior gonad arm is on the right side of the animal with the posterior arm on the left, (*d*) complete gut/gonad heterotaxy: the positions of the anterior and posterior gonad arms and gut are L/R-reversed, whereas the anterior pair of coelomocytes remains on the right, (*e*) posterior heterotaxy: the position of only the posterior gut and gonad is reversed compared to that in dextral hermaphrodites, (*f*) anterior heterotaxy: the position of only the anterior gut and gonad is reversed compared with that in dextral hermaphrodites.

**Figure 2.**
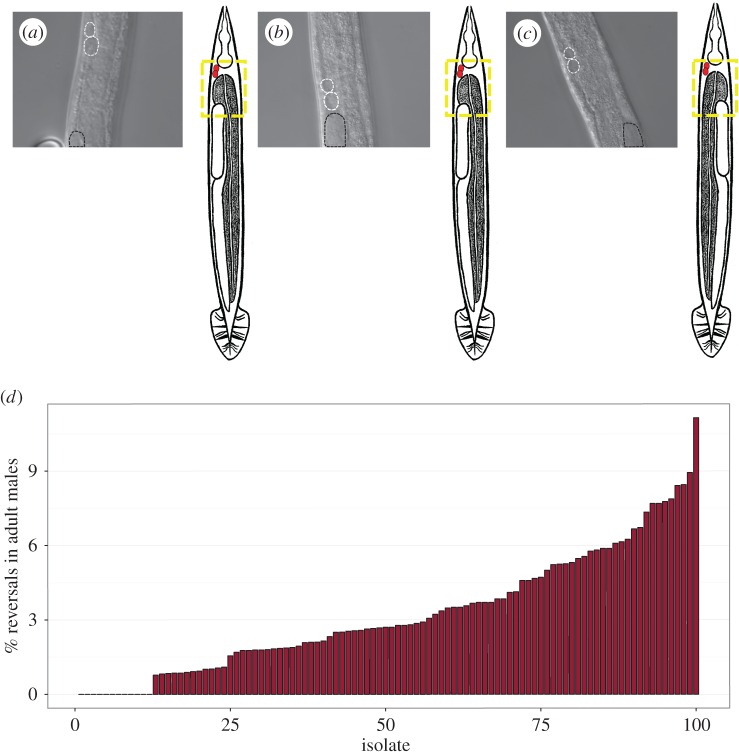
The propensity for L/R gut/gonad reversals varies widely in males of 100 *C. elegans* isolates. (*a–c*) Examples of DIC images of ventral view showing the relative position of the gonad. Coelomocytes are marked with white-dashed lines and the top of the gonad arm is marked with black dashed lines. Dotted yellow box in the adjacent cartoon denotes the corresponding region in the photomicrograph in reference to the rest of the body. (*a*) Dextral N2 male, (*b*) dextral male from isolate JU778, (*c*) reversed JU778 male. (*d*) Percentage L/R gut/gonad reversals in adult males in the different natural isolates grown at 20°C. Each isolate is numbered 1–100 in rank order based on reversal frequency observed. Sample size per isolate ranges from 90 to 700 animals (a single replicate per isolate, except for isolates AB4, QX1211, N2 and CB4856, for which three replicates were performed and data were pooled when *p* > 0.05 using Fisher's exact test). See electronic supplementary material, table S1 for the key to the numbered isolate list and additional information.

We previously reported that the L/R gut/gonad asymmetry in N2 males raised at 25°C is frequently (more than 5%) reversed [40], with the fully developed gut occupying the position on the right normally taken by the gonad (figure 1). These reversals do not appear to reflect a complete inversion of L/R anatomy, as a marker of the right-specific ASER neuron is expressed in its normal position on the right [34]. We also observed reversed males in the Hawaiian (Hw) isolate CB4856 and in recombinant inbred advanced intercross lines (RIAILs) derived from N2/Hw crosses [43,44] under standard conditions, suggesting that this effect does not simply reflect breakdown of the L/R-determining system for these organs as a result of long-term culturing in the laboratory. These findings led us to ask whether such reversals might also occur naturally in the many available naturally inbred wild isolates of the *C. elegans* species, which show variation in a number of other traits, including vulval development [45,46], susceptibility to pathogens [47,48] and anti-parasitic resistance [49]. Indeed, we found that among 100 wild isolates, there is widespread variation in the propensity for gut/gonad reversals in adult males: while 12 isolates never showed reversals (electronic supplementary material, table S1, *n* > 90 per isolate), most isolates showed significant rates of reversals with frequencies as high as 11.2% (JU778, *n* = 35/314; figure 2*d*; numerical data and confidence intervals shown in the electronic supplementary material, table S1). Thus, although *C. elegans* development is generally seen to be highly stereotyped, the L/R arrangement of the major organs in males is substantially variable in many wild isolates of the species (observed for a total of 474 out of 13 712 males).

### (b) Frequent reversals in male gut/gonad asymmetry occur in both hermaphroditic and gonochoristic *Caenorhabditis* species

**Figure 3.**
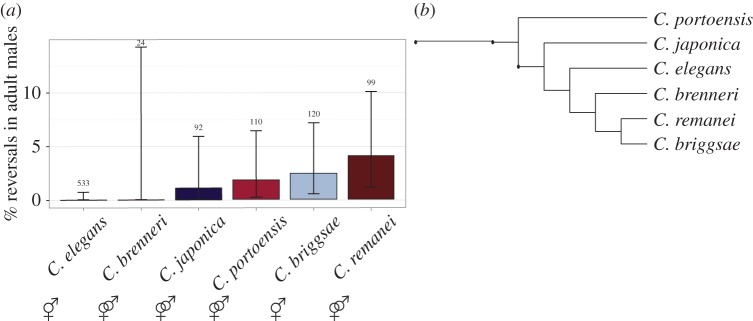
Other *Caenorhabditis* species show gut/gonad reversals in males. (*a*) L/R gut/gonad reversals in males of the indicated species. Black bars represent 95% binomial proportion confidence intervals calculated using the Clopper–Pearson/exact method. Sample size is noted above each confidence interval (*n* = 1 replicate per isolate). Reproduction mode (gonochoristic or hermaphroditic) is indicated below each bar. (*b*) Abbreviated phylogenetic tree shows relationship of select *Caenorhabditis* species based on [50].

We considered the possibility that natural variation in male L/R development of *C. elegans* isolates might be the result of reduced selective pressure on the male body plan in the species, which is hermaphroditic and can therefore effectively propagate in the absence of males. Consistent with such a possibility, we found that males of the hermaphroditic species *C. briggsae* also showed frequent L/R reversals (figure 3). However, we also found that three of four closely related gonochoristic *Caenorhabditis* species (figure 3) analysed, *C. japonica*, *C. remanei* and *C. portoensis*, showed frequent gut/gonad reversals in males, even under conditions (20°C) in which N2 males never showed such reversals (figures 2*d* and 3*a*). Thus, the substantial variation in L/R orientation of the major organs is common in males of *Caenorhabditis* species, even when sex is essential for their propagation.

### (c) Extensive variation in left–right orientation of each gonad arm in hermaphrodites

**Figure 4.**
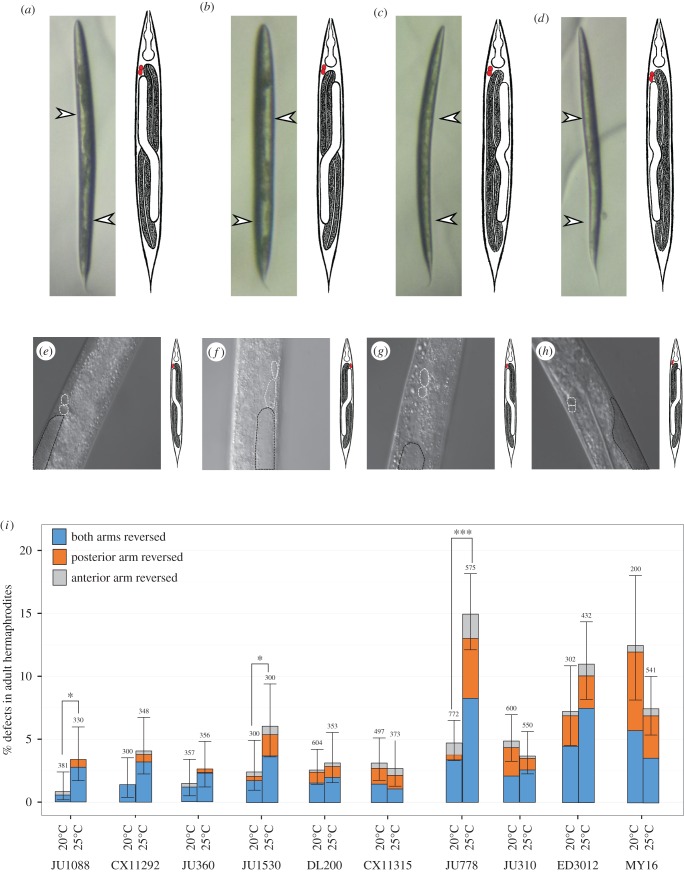
Temperature effect on hermaphrodite heterotaxy. (*a–d*) Ventral view of hermaphrodites (anterior up). White arrows denote position of gonad arms, which are visible as clear patches under the dissecting scope. Adjacent cartoons denote the type of heterotaxy observed, as described in figure 1. (*a*) Dextral hermaphrodite from isolate JU778. (*b*) Reversed hermaphrodite from isolate JU778. (*c*) Posterior heterotaxy from isolate JU778. (*d*) Anterior heterotaxy from isolate JU778. (*e–h*) Higher magnification DIC images showing the anterior coelomocytes ccAR and ccPR (white dashed lines) and anterior portion of gonad arm (black dashed lines; N2). (*e*) Dextral hermaphrodite from isolate N2. (*f*) *gpa-16(it143)* hermaphrodite with fully sinistral handedness (*situs inversus totalis*). Note that both the anterior gonad arm and the coelomocyte pair ccAR and ccPR are on the left side. (*g*) Dextral hermaphrodite from isolate JU778. (*h*) Complete heterotaxy in hermaphrodite from isolate JU778. The coelomocyte pair ccAR and ccPR remain on the right side of the animal, but the gut and gonad positions are reversed. (*i*) Frequency of each class of heterotaxy in hermaphrodites from a subset of *C. elegans* isolates cultured at 20°C and 25°C. Black lines represent 95% binomial proportion confidence intervals calculated using Clopper–Pearson/exact method. Pooled sample size is noted above each condition (*n* ≥ 3 replicates per isolate). Fisher's exact test was used to compare frequencies of heterotaxy at 20°C and 25°C for each isolate. (*p < 0.05, **p < 0.01, ***p < 0.001.)

Given our finding that males of both hermaphroditic and gonochoristic species exhibit L/R gut/gonad reversals, we asked whether similar variation in the relative position of the internal organs might also occur in hermaphrodites. In contrast to the single-arm male gonad, the hermaphrodite gonad consists of two arms arranged in opposite anterior–posterior orientation (figures 1*c* and 4*a*). Each arm terminates in a spermatheca, both of which connect to a common uterus. In both sexes, the four-cell gonad primordium is present mid-ventrally in the newly hatched L1 larvae; however, the postembryonic lineages and development differ substantially in the two sexes [41,51,52]. Given that natural reversals were never observed in a large number of hermaphrodites (more than 15 000) in the N2 isolate [17,35,42], it is possible that coordinating the migration of two gonad arms might require a more robust and high-fidelity developmental programme than in males. However, although we found that several isolates never showed reversals in hermaphrodites (seven isolates, *n* > 100 each, greater than or equal to two replicates per isolate), hermaphrodites from some isolates showed frequent gut/gonad reversals, ranging from 0.8% (*n* = 381) in isolate JU1088 to 12% (*n* = 200) in MY16 at 20°C (figure 4). Thus, natural reversal in the L/R orientation of the major organs is a characteristic of both sexes.

The gut/gonad reversals that we observed in hermaphrodites frequently involved only a single gonad arm. In normal N2 hermaphrodites, the anterior arm resides on the right and the posterior on the left, when viewed from the ventral aspect (figure 3*a,e*). We observed three classes of gut/gonad reversals in hermaphrodites: those involving only the anterior arm (‘anterior heterotaxy’), only the posterior arm (‘posterior heterotaxy’) or both arms (‘complete reversal’), in which the overall orientation of the gut and gonad is a mirror image of that in a normal dextral hermaphrodite (figure 3*b,c,d,g,h* and figure 1*c–f*). The frequency for each type of defect varied from 0% to 6% at 20°C (*n* > 200 for each isolate; figure 4*i*). Reversals involving only one arm (figure 4*c,d,g,h,i*) were substantially more prevalent than those involving both, suggesting that the event leading to these reversals may occur independently during gonad development.

All classes of gut/gonad reversals in hermaphrodites of several of the isolates were significantly more frequent at 25°C than at 20°C (e.g. as was particularly evident for isolates JU1088, JU1530, and JU778; Fisher's exact test, *p* < 0.05, *p* < 0.05, *p* < 0.001 respectively, figure 4). This might indicate inherent temperature sensitivity in the process of gonad migration, possibly akin to the temperature sensitivity of the nuclear migration of the ventral P epidermal cells during early larval development [53]. However, some isolates showed no significant enhancement in the rate of L/R gut/gonad reversals (and in some cases, showed a lower rate of reversals at the higher temperature, albeit at insufficient sample size to provide statistical significance), suggesting a more complex and varied relationship between establishment of this L/R asymmetry and temperature.

### (d) Heterotaxy is independent of the event that establishes embryonic chirality and overall anatomical handedness

The overall anatomical L/R asymmetry is established in the early embryo, when the mitotic spindles of the daughters of the AB blastomere skew along the A/P axis with a dextral bias [18,32,35,54]. While this event leads to the defined dextral anatomical handedness of the animal, L/R anatomical handedness can be completely reversed (i.e. *situs inversus totalis*) by micromanipulation of the skewing spindles [35] and in *gpa-16* mutants, in which the orientation of the mitotic spindle is altered in the early embryo [17]. To assess whether the reversals we observed reflect reversals in the chirality-inducing event in the early embryo, we took advantage of the reproducible L/R asymmetry in the position of the coelomocytes, which can be scored in living animals by Nomarski microscopy. We analysed 10 isolates for association of L/R reversals with overall anatomical handedness, using the ventral pair of coelomocytes ccAR and ccPR as an internal marker of the overall chirality of the animal [42]. In hermaphrodites showing *situs inversus totalis* as a result of a temperature-sensitive mutation in *gpa-16(ts),* the anterior pair of coelomocytes (ccAR and ccPR) invariably (*n* = 10) are located on the left instead of at their normal position on the right, and the gut/gonad arrangement is sinistral (figure 3). In contrast, for all 10 isolates examined, the coelomocyte pair was invariably located in the normal right-side position, including in all animals with partial or complete gut/gonad reversals (*n* > 43 animals with reversals; figure 3). These observations strongly suggest that reversal in the position of the gut and gonad occurs independently of the overall anatomical chirality established in the early embryo and hence reflects *bona fide* heterotaxy of the major organs.

### (e) An event preceding mid-embryogenesis can influence heterotaxy much later in development

**Figure 5.**
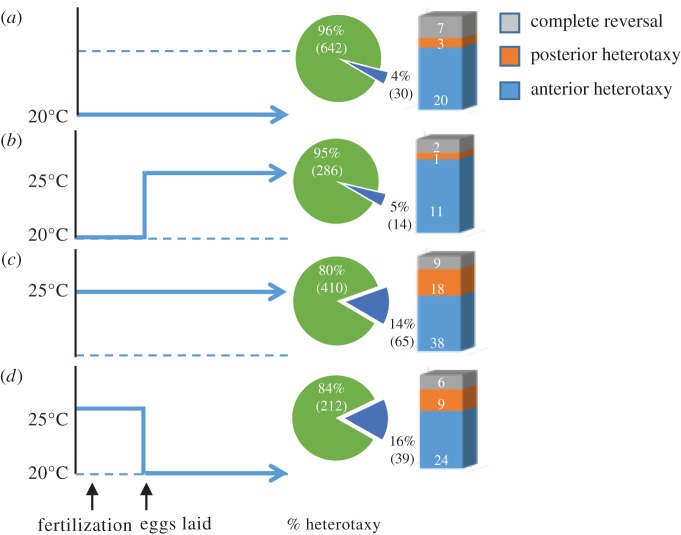
Temperature-sensitive period influencing heterotaxy coincides with *in utero* embryonic development. Adjacent piecharts show proportion of hermaphrodites with any heterotaxy and stacked bar shows proportion of each class of heterotaxy. (*a*) Pre-gastrulation-stage embryos cultured continuously to adulthood at 20°C (*n* = 672). (*b*) Pre-gastrulation-stage embryos upshifted from 20°C to 25°C and cultured to adulthood at 25°C (*n* = 300). (*c*) Pre-gastrulation-stage embryos cultured continuously to adulthood at 25°C (*n* = 475). (*d*) Pre-gastrulation-stage embryos downshifted from 25°C to 20°C and cultured to adulthood at 20°C (*n* = 251). Sample size is pooled from *n* ≥ 2 replicates per condition when *p* > 0.05 using Fisher's exact test. Fisher's exact test was also used to compare all experimental conditions.

Our observation that the propensity for L/R reversals in gut and gonad arrangement is influenced by temperature led us to evaluate the developmental stage at which the sensitivity to temperature for this effect occurs in hermaphrodites of isolate JU778, which shows among the highest rates of reversals. Synchronized populations of JU778 animals were grown at either 20°C or 25°C for at least two generations and early pre-gastrulation embryos, present within the uterus of hermaphrodite adults, were temperature-shifted up or down and scored for heterotaxy (figure 5). JU778 hermaphrodites maintained at 20°C or those upshifted during gastrulation to 25°C (figure 5*a,b*) showed similarly lower rates of heterotaxy (4.5%, *n* = 672 and 4.7%, *n* = 300, respectively; Fisher's exact test, 5% false discovery rate [55], *q* = 0.87). In contrast, animals grown continuously at 25°C (13.7% reversals, *n* = 475), and pre-gastrulation embryos that were downshifted from 25°C to 20°C (15.54%; *n* = 251) showed similarly (Fisher's Exact Test, 5% false discovery rate [55], *q* = 0.61) higher rates of heterotaxy (figure 5*c*) compared with those grown at 20°C (*q* < 0.0001, Fisher's exact test; figure 5*d*). This finding suggests that the cellular events leading to L/R reversals in response to temperature occur during early embryogenesis or perhaps in the maternal germline, long before the migration of the DTCs that occurs during the larval stages.

### (f) Genome-wide association identifies three major regions associated with male left/right gut/gonad reversals

**Figure 6.**
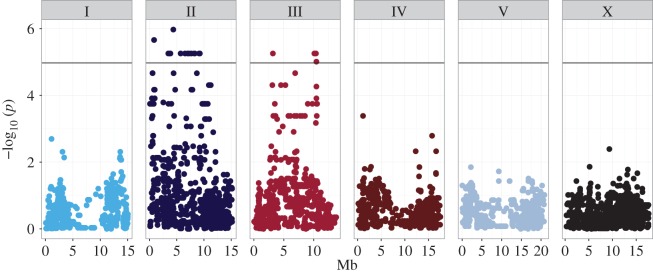
Genome-wide association using efficient mixed-model analysis (EMMA) identifies three major genomic regions that are significantly associated with reversals in adult males across *C. elegans* isolates. −log_10_ of *p*-values for each SNP were calculated using likelihood ratio tests and maximum-likelihood estimation. To control for multiple comparisons, a Bonferroni cut-off was used (*α* = 0.05/4690 = 4.97 × 10^−5^) and is represented by the black line. Genome locations are represented on the *x*-axis, chromosomes indicated by the numbered boxes (I–V, X), and position along the chromosome is represented in mega base pairs (Mb) on the *x*-axis. See electronic supplementary material, figure S1.

In an effort to characterize the genetic complexity leading to heterotaxy in males, we performed a genome-wide association study (GWAS) using linear mixed-model analysis, including a pairwise identity-by-state (IBS) kinship matrix to correct for population structure [56], based on the distribution of this phenotype across 100 wild isolates (figures 2 and 6). Owing to the highly variable nature of this phenotype (figure 2, electronic supplementary material figure S1 and table S1), we initially compared the frequency of reversals for all 100 natural isolates against each other before association mapping. The majority of significant comparisons were found between isolates at the extreme ends of the phenotypic spectrum, where the only significant comparisons were between isolates with 0% and approximately 8–11% L/R reversals (Fisher's exact test, 5% false discovery rate [55], *q* ≤ 0.049). In order to identify any loci associated with the propensity for heterotaxy, we chose to treat the phenotype as a binary, categorical variable and used this category for association mapping. Thus, to perform the GWAS, we collected the 12 isolates with reversal frequencies of 0% into one group (electronic supplementary material, table S1) and the remaining 88 isolates into another.

By applying this binary/categorical phenotype, we found that GWAS identified one highly significant major region on chromosome II (23 SNPs) and two significant regions on chromosome III (nine SNPs total). The region of chromosome II spans 10.75 Mbp (0.55 Mbp to approx. 11.3 Mbp), and all SNPs significantly associated with the phenotype in this region were found to be in near-perfect linkage disequilibrium (LD; *R*^2^ = 0.88) [57]. The first peak on chromosome III spans a 4.3 Mbp region (2.61–6.91 Mbp) and all significantly associated SNPs also show moderate LD (*R*^2^ = 0.58). The second peak on chromosome III spans a small, 0.4 Mbp region (10.1 Mbp to approx. 10.5 Mbp), which is also in nearly perfect LD (*R*^2^ = 0.75), but only in moderate LD with the other region on chromosome III (*R*^2^ = 0.53). Although the region on chromosome II and first region on chromosome III have sufficiently large recombination intervals (19.1 cM on chromosome II, first region on chromosome III approx. 10.29 cM) that there would, in principle, be expected to be frequent recombination events separating the causal loci from linked SNPs, it has been shown that *C. elegans* natural isolates have recently undergone selective genomic sweeps and hence share large genomic segments [58]. Thus, rare variants affecting the variability of heterotaxy that have accumulated in these regions may be inherited as large shared blocks that include the causal loci.

We sought to estimate heritability of this trait [59–62] by analysing the genetic and random (owing to measurement error) variance for each of 4690 SNPs using restricted maximum-likelihood method (REML). We included an IBS kinship matrix in the REML estimates to control for population structure. This analysis yielded a heritability (*h*^2^) of 0.302. However, this estimate may be slightly biased and/or artificially inflated: as interaction and dominance terms cannot be parsed from GWAS alone, our analysis considers only additive effects for each SNP. Moreover, heterotaxy is significantly affected by temperature with many isolates (figure 4 and electronic supplementary material, figure S1), indicating an environmental component or genotype–environment interaction that is not included in our heritability estimate (figure 6).

### (g) The major variation in heterotaxy between an isolate with no reversals and one with frequent reversals is attributable to a small number of loci

**Figure 7.**
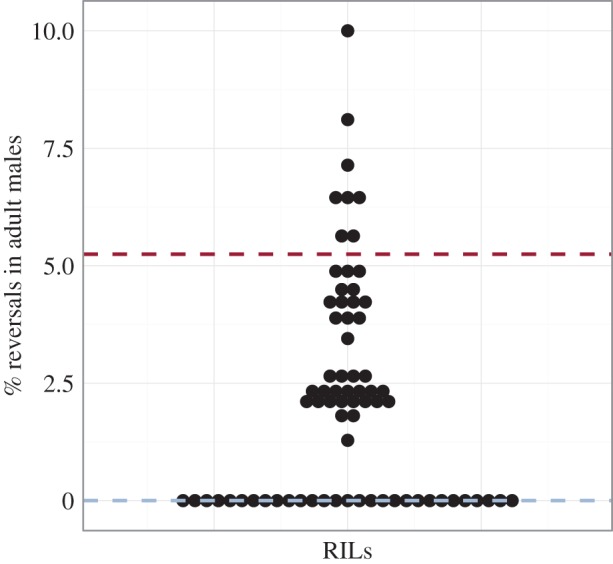
Frequency of reversals in males of RILs. Each of 72 RILs is represented by a dot. The *y*-axis corresponds to % reversals. Parental isolates are marked by dashed lines (AB4 in blue and QX1211 in red). Sample size varies from 30 to 100 males per RIL (*n* = 1 replicate per RIL).

The GWAS analysis suggested that there are at least three loci that influence the prevalence of heterotaxy across the 100 wild isolates. However, GWAS identifies only those genomic regions that vary in a substantial number of isolates. We sought to assess the genetic complexity of this trait further by analysing its segregation following a cross between an isolate with no observed heterotaxy (AB4) and one that shows a high incidence for the trait (QX1211). Recombinant inbred lines (RILs) [63,64] were generated from this cross and the frequency of L/R gut/gonad reversals in males was analysed (figure 7 and electronic supplementary material, figure S4 and table S2). Of 72 RILs, 29 (approx. 40%) showed no reversals, suggesting relatively low genetic complexity for the trait. The remaining RILs showed reversal frequencies of up to 10%, with no strong evidence of transgressive segregation (Fisher's exact test, 5% false discovery rate [55], *q* > 0.05). These results indicate that heterotaxy is a genetically tractable trait for which there may be one or a small number of loci underlying the phenotype in the QX1211 isolate; however, further analysis will be required to determine the effect of each locus *in vivo.*

## 3. Discussion

We report five major advances that reveal both the complexity of events leading to L/R asymmetry in the arrangement of organs in a simple metazoan, and the evolutionary plasticity underlying it. (i) We found that, across a single species with a generally highly determinate development, the stereotyped arrangement of the two major organs, the gut and gonad, is subject to wide variation in fidelity across wild isolates in both sexes: while some naturally inbred isolates never reverse the canonical arrangement, other isolates show frequent L/R reversals, without reversing the overall chirality of the animal established during early embryogenesis. Thus, these reversals reflect *bona fide* heterotaxy. (ii) L/R reversals can affect either the anterior or posterior arm of the hermaphrodite gonad, suggesting that the symmetry-breaking mechanism that affects the arrangement of these organs may be assessed independently for each domain of the organ. (iii) Frequent reversals are also observed in males of other nematode species, including those in which males are obligate for reproduction, implying that a tendency towards heterotaxy is not the result of relaxed selection of male function in hermaphroditic species. (iv) The propensity for heterotaxy varies with temperature and the temperature-sensitive period for this effect in one isolate occurs unexpectedly early, well before the stage at which the reversals are observable. Thus, an event in the very early embryo or maternal germline influences a symmetry break that is evident only much later in development. (v) Variation in the propensity for heterotaxy across the 100 isolates is attributable, in part, to differences in three regions of the genome, and a small number of genetic changes appear to account for the variation between one isolate with high rates of heterotaxy, and one that shows none. Thus, the variation in the frequency of heterotaxy in *C. elegans* may be a relatively simple genetic trait.

Our finding that heterotaxy is common in many wild isolates suggests that, although the overall embryonic chirality and a number of L/R asymmetries in the animal appear to be obligately rigidly determinate, the specific L/R orientation of the two largest internal organs is not. Such naturally occurring heterotaxy in *C. elegans*, exceeding a rate of 10% in some natural isolates, is substantially greater than the prevalence of heterotaxy for the human population, which has been estimated to be one in 15 000 [65]. However, it is interesting to note that, as we have observed with different isolates of *C. elegans*, the prevalence of heterotaxy varies substantially between different groups of humans, [28], with a higher prevalence among individuals of Asian descent. Thus, the developmental fidelity of L/R organ packing is subject to substantial variation across different populations of these phylogenetically widely separated species.

In contrast with males, in which the gut/gonad orientation is either normal or fully reversed, reversals of the L/R arrangement of the anterior and posterior hermaphrodite gonad arms appear to be independently determined. It is not clear whether the uncorrelated heterotaxy between the two arms reflects an L/R symmetry-breaking process that occurs independently for both arms, or a single system that influences the probability that each arm will occupy its normal or reversed orientation. In contrast, there appears to be some correlation between the prevalence of heterotaxy in males and hermaphrodites in the most strongly affected isolates. This correlation is not absolute, however, as some isolates that showed substantial heterotaxy in hermaphrodites (JU360, JU1088, ED3012) did not show male heterotaxy at 20°C. It will be of interest to assess the role of sex-determining mechanisms in influencing the L/R handedness-determining mechanisms of the major organs.

We found that the frequency of organ reversals is temperature-dependent in many of the wild isolates, indicating that there is an environmental component to the observed heterotaxy. Temperature-induced stress is widely known to lead to errors in development ([66] and reviewed in [67]), and could explain the tendency towards increased heterotaxy in these isolates. However, this cannot fully account for the observed effect of temperature, as some isolates showed an indication of reduced heterotaxy at elevated temperatures. While the L/R heterotaxy we have observed in both sexes of *C. elegans* is evident well after embryogenesis, we were surprised to find that the sensitive period for the temperature-dependent event that influences the frequency of heterotaxy is during very early embryogenesis or even in the maternal germline. Thus, an event preceding organogenesis or even any overt differentiation affects a symmetry-breaking process that occurs only after the gut and gonad organs are formed and undergo maturation during larval development. It is conceivable that a chiral structure in the oocyte or early embryo, perhaps an element of the cytoskeleton, might establish an L/R asymmetry that is interpreted much later at the time that the distal end of the gonad begins to migrate, leading to elongation of the gonad arm.

**Figure 8.**
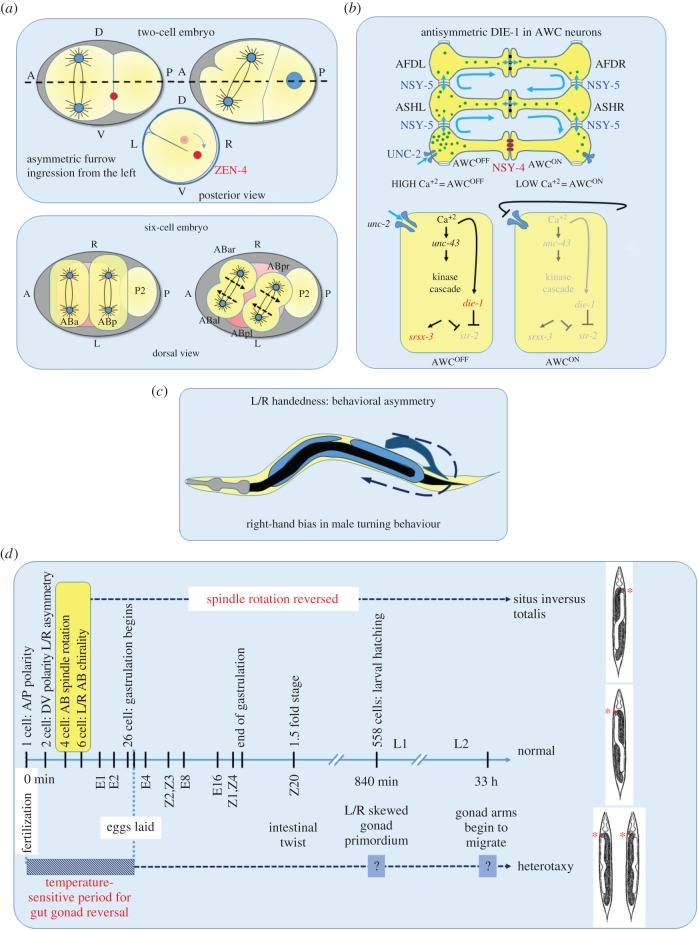
L/R developmental and behavioural asymmetries in *C. elegans*. (*a*) Actomyosin cortical flow-driven bilateral asymmetry in the early embryo. The one-cell embryo exhibits a dextral rotation within the eggshell as a result of chiral flows generated by cortical actomyosin contractility [54]. The chirality is maintained in the AB cell, resulting in L/R differences in the cortex, which is manifested in asymmetric cleavage furrow initiation from the left side [18,69]. During the transition from the four- to six-cell stage, the torque generated by counter-rotating cortical flows in the two AB daughter cells results in skewing of the spindles, resulting in a chiral arrangement of the four granddaughters of the AB blastomere [18]. This chirality establishes most of the later L/R anatomical asymmetry of the animal [35]. (*b*) Calcium signalling through gap junction coupled cell network biases asymmetric neuronal fate. The L/R differences between the two, functionally distinct, bilateral AWC neurons are stochastically determined. High-calcium signalling in AWC autonomously promotes AWC^OFF^ [70–72] fate via the expression of the transcription factor DIE-1 [73], whereas calcium signalling in the non-AWC NSY-5 gap junction cell network promotes AWC^ON^ fate. In 50% of the animals, AWC^ON^ is on the right and AWC^OFF^ on the left; in the other 50%, their positions are reversed. (*c*) Bilaterally asymmetric motor behaviour. Males exhibit a right-hand turning bias that is independent of L/R anatomical asymmetry and embryonic chirality [24]. (*d*) Gut/gonad heterotaxy: L/R embryonic chirality establishes asymmetric cellular interactions that result in bilateral differences in cell and organ positions. In normal (dextral) worms, the anterior coelomocytes (marked by asterisks) and the anterior gonad arm of the hermaphrodites and entire gonad in males is on the right, whereas the gut is on the left. Reversal of spindle skewing of the AB daughter cells results in animals with completely reversed handedness (*situs inversus totalis*) [35]. The gut/gonad reversals (anterior and posterior heterotaxy) observed in many natural isolates shows temperature sensitivity during early embryonic development, which affects L/R asymmetric migration of gonad arms later during larval development. They are independent of the initial L/R symmetry-breaking events in the embryo.

A recurring theme emerging from recent studies on *C. elegans*, Xenopus and gastropods is that establishment of L/R differences in gene expression, and overall handedness, depends on the regulation of cytoskeletal rearrangements across these divergent organisms [68]. However, it is unclear whether the particular pathways that regulate the cytoskeleton to direct L/R symmetry breaks are phylum-specific. In *C. elegans*, the actomyosin cortex plays a critical role in establishing the overall L/R anatomical asymmetry [17,18,35,54] by generating chiral counter-rotating cortical flows along division axes, thereby generating torque that causes the spindles to skew clockwise during formation of the six-cell embryo (figure 8*a*). This initial bilaterally asymmetric arrangement of the blastomeres establishes bilateral differences in lineages, fates and position of cells that persist into adulthood [34,35,74].

Might cytoskeletal rearrangements also influence symmetry-breaking events that occur later in development, including those that underlie the type of heterotaxy we have described here? Recently, Davison *et al*. [75] reported that the L/R axis in Xenopus and snails is most probably determined by the regulation of cytoskeletal elements that are predetermined and inherent to the chirality of the embryo itself [32,76,77]. Further, it was reported that heterotaxy in Xenopus and chirality of snails could be caused by ectopically expressing formin, which regulates polymerization of the actin cytoskeleton. It may be noted that of at least seven *C. elegans* genes that encode proteins with formin-like domains, four (*exc-6*, *frl-1*, *fozi-1* and *cyk-1* [78–82]) reside on chromosome III, on which we have identified two significant regions associated with heterotaxy. However, our initial genetic analysis is of insufficient resolution to point to specific causal mediators of gut/gonad heterotaxy. Further studies, using information from the RILs, will make it possible to delineate quantitative trait loci (QTL) that underlie heterotaxy in both sexes. It will be of interest to learn whether the causal genetic variations leading to heterotaxy in *C. elegans* play conserved roles in L/R organ arrangement in other bilateria and whether there is any relationship between them and the L/R randomization of internal organs seen in humans, for example in Kartagener's syndrome [29].

Although handedness asymmetry in the arrangement of the visceral organs is particularly striking, a number of other L/R asymmetries in vertebrates, including brain laterality, ocular dominance and motor handedness [83–86], are established by mechanisms that are largely (though not fully) independent during development. The findings reported here lend further support to the view that a multiplicity of independent symmetry-breaking events lead to distinct L/R asymmetries even in a very simple animal with less than 1000 somatic cells (summarized in figure 8). The first and major symmetry break, occurring at the 4–6 cell stage of embryogenesis, sets the stage for many subsequent L/R differences in cell positions and fates throughout the rest of development. However, a number of later symmetry-breaks that are independently assessed occur with particular cell types and behaviours. Stochastic establishment of asymmetric cell identities in the nervous system resulting from cellular interactions that are independent of the early chirality-establishing events occur during specification of the bilateral pair of AWC neurons: i.e. asymmetric calcium signalling mediated by innexins, claudins [73,87] and the transcription factor DIE-1, establishes asymmetric expression of chemoreceptors in AWC neurons (figure 8*b*) [70,73]. Further, *C. elegans* males exhibit a right-handed motor behaviour bias during mating that is also independent of anatomical L/R asymmetry [24] (figure 8*c*), implying the existence of yet another L/R symmetry-breaking process that affects motor output. The frequent heterotaxy we have observed suggests that there may exist yet another symmetry-breaking mechanism that affects the L/R arrangement of the major organs in the animal independent of embryonic chirality (figure 5*d*). That several, apparently independent, processes function to direct L/R differences throughout development in this simple animal underscores the critical importance of distinguishing differences in bilateral structures and activities in many contexts during the formation of bilaterian metazoans.

## 4. Material and methods

### (a) General maintenance and isolates used

Except where noted *C. elegans* isolates and *Caenorhabditis* species were maintained as described by Brenner [88] and scored at room temperature. BW1089—*gpa-16(it143)* I; *him-5(e1490)* V. The isolates corresponding to different *Caenorhabditis* species used in this study are N2—*C. elegans*, CB5161—*C. brenneri*, JU724—*C. remanei*, DF5081—*C. Japonica*, EG4788—*C. portoensis*, AF16—*C. briggsae*. For a list of *C. elegans* isolates used in this study, see electronic supplementary material, table S1.

### (b) Generation of male worms

To obtain males, 20–30 L4 hermaphrodites were picked into 7% ethanol solution in microcentrifuge tubes and rotated for an hour (modified from [89]). Worms were pelleted by centrifugation at 2000 rpm for 10 s. They were then transferred to fresh nematode growth medium (NGM) plates seeded with *E. Coli* strain OP50 and incubated at 20°C. F_1_ male progeny were mated with sibling hermaphrodites to establish male stocks.

### (c) Generation of recombinant-inbred lines

Males from isolate QX1211 were crossed with sperm-depleted hermaphrodites from isolate AB4. Thirty F_1_ progeny were allowed to self-fertilize, and 150 F_2_ progeny were individually cloned and allowed to self-fertilize for 10 generations before freezing [90]. While many lines underwent attrition during propagation, 84 RILs were successfully generated.

### (d) Scoring gut and gonad orientation

The gut/gonad orientation in males was performed as described previously [40]. Briefly, 20–30 adult worms were anaesthetized in 3 µl of 5 mM levamisole on a 5% agarose pad. An eye-lash tool was used to orient the males ventral-side up, and slides were then sealed. Mounted worms were imaged at 10× and 63× magnification on a Zeiss Axioskop 2.

Hermaphrodite gut/gonad orientation was scored on unseeded NGM plates by allowing L4 or young adults to chemotax towards a spot of OP50 *E*. *coli* mixed with 1 M sodium azide for 1 h at room temperature. Anaesthetized worms were then rolled using an eye-lash tool and scored ventral side up on a Motic dissecting scope and imaged at 45×.

### (e) Temperature-shift experiments

Continuously fed stocks of JU778 were grown at 20°C or 25°C for 7 days (approx. two to three generations) before temperature shifts. Synchronized L1 larvae were collected by hatching embryos from gravid adults treated with alkaline hypochlorite solution and then grown on NGM plates seeded with OP50 at 20°C or 25°C until they were gravid adults but before any eggs were laid on the plates. They were then lysed, using alkaline hypochlorite solution [91] and the surviving pre-gastrulation embryos were collected. Embryos were then placed on NGM plates with food and allowed to develop at 20°C or 25°C. Gut/gonad orientation was then scored as described above.

### (f) Statistical analysis

Data analyses were performed with R statistical software v. 3.2.3. Fisher's exact tests were used to compare frequencies between isolates, unless otherwise stated. All binomial proportion confidence intervals were calculated, using the Clopper–Pearson/exact method with the R package binom and the function binom.confint() (electronic supplementary material, tables S1 and S2 and figure 3, figures S1, S3 and S4). The false discovery rate method (*q*-value) developed by Benjamini & Hochberg [55] was used to correct for multiple comparisons for all tests excluding GWAS.

### (g) Genome-wide association study analysis

GWAS analysis was performed using the EMMA package for R [56]. Genotype information used was a binary SNP map with 4690 markers spaced by approximately 100 kb per marker [58]. Likelihood ratio tests were used to determine *p*-values per SNP using the emma.ML.LRT() function. Mixed-model association tests also included an IBS kinship matrix calculated using the binary SNP map, which accounts for relatedness and population structure [56], and were performed using the emma.kinship() function in the EMMA package for R. The Bonferroni method was used to account for multiple comparisons. The black line spanning all chromosomes in figure 7 represents the significance threshold *α* = 0.05/4690 = 4.97 × 10^−5^. The phenotypic input for mixed modelling was binary: isolates with 0% reversals were assigned to an arbitrary group 0, and all other isolates were assigned to an arbitrary group 1. 10 000 permutations of *p*-values were obtained from GWAS without accounting for population structure in order to confirm that the *p-*values for significant SNPs (*n* = 32) were not attributable to random chance alone (electronic supplementary material, figure S1). LD for the three loci identified by EMMA was calculated using PLINK software v. 1.07 [92].

### (h) Heritability estimate

To estimate genome-wide heritability (*h*^2^), we used a mixed-model approach that also allowed us to account for population structure and derive empirical variance estimates for each SNP [56]. To estimate relatedness between isolates and control for population structure, we used an IBS kinship matrix and REML to calculate the genetic and random variance components for each SNP using the emma.REML.t() function. We then calculated heritability as 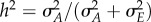 [62].

## Supplementary Material

Heterotaxy in Caenorhabditis: supplementary material The propensity for L/R gut-gonad reversals varies widely in males of C. elegans isolates at 20°C.
