# Supplementary material for: Heterotaxy in Caenorhabditis: widespread natural variation in left–right arrangement of the major organs
Source: Philos Trans R Soc Lond B Biol Sci. 2016 Dec 19;371(1710):20150404. doi: 10.1098/rstb.2015.0404 (PMC5104504; doi:10.1098/rstb.2015.0404)
Supplement: Heterotaxy in Caenorhabditis: supplementary material The propensity for L/R gut-gonad reversals varies widely in males of C. elegans isolates at 20°C. [file rstb20150404supp1.pdf]

Supplementary material for [Alcorn, MR et al], [2016], [Heterotaxy in Caenorhabditis: widespread natural variation in L/R arrangement of the major organs], *Phil. Trans. R. Soc. B.* doi: 10.1098/rstb.2015.0404

Supplementary Figure 1: The propensity for L/R gut-gonad reversals varies widely in males of *C. elegans* isolates at 20˚C. In figure 2, isolates are numbered in ascending order. This figure includes lower and upper bounds for 95% Binomial proportion confidence intervals calculated using the Clopper-Pearson/Exact method (See Materials and Methods).

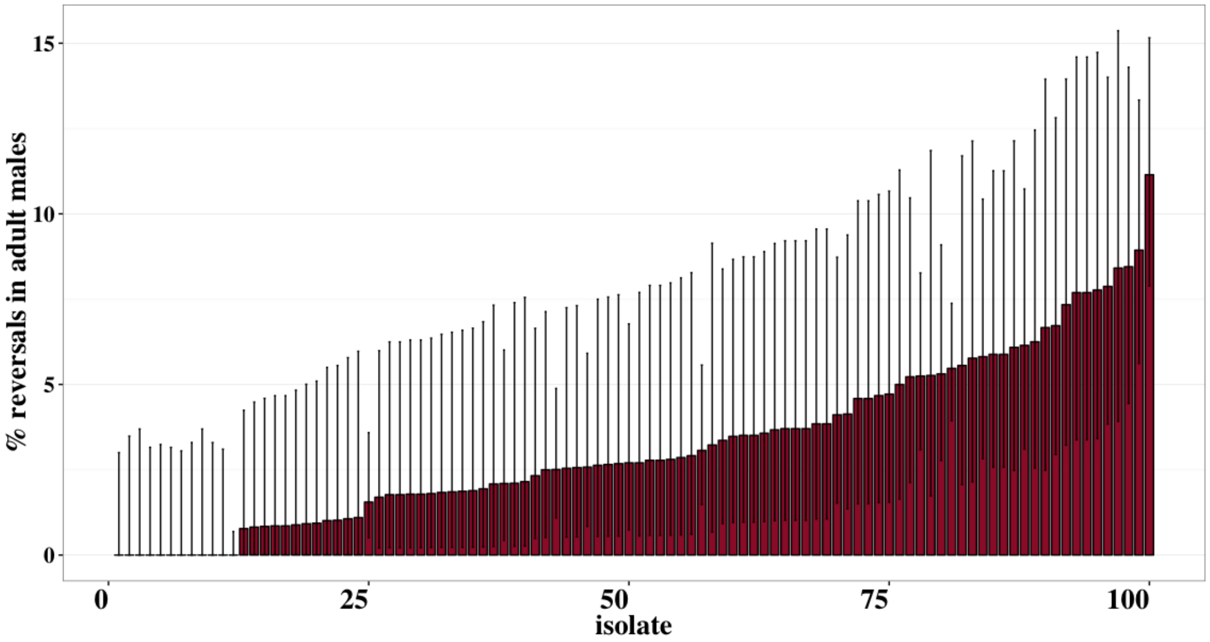

Supplementary Table 1: Complementary data for Figure 2: The propensity for L/R gut-gonad reversals varies widely in males of *C. elegans* isolates at 20˚C. In figure 2, isolates are numbered in ascending order. This table includes isolate name, total sample size, percent reversals and the lower and upper bounds for 95% Binomial proportion confidence intervals which were calculated using the Clopper-Pearson/Exact method (See Materials and Methods). The full data set including confidence intervals is depicted in Supplemental Figure 1.

Isolate Number	Isolate	Total Sample Size	% Reversals	Lower Bound %	Upper Bound %
**1**	AB4	121	0.00	0.00	3.00
**2**	CB4857	104	0.00	0.00	3.48
**3**	CX11292	98	0.00	0.00	3.69
**4**	ED3012	115	0.00	0.00	3.16
**5**	JU1088	112	0.00	0.00	3.24
**6**	JU1440	115	0.00	0.00	3.16
**7**	JU1530	119	0.00	0.00	3.05
**8**	JU360	110	0.00	0.00	3.30

© The Authors under the terms of the Creative Commons Attribution License http://creativecommons.org/licenses/by/3.0/, which permits unrestricted use, provided the original author and source are credited.

**9**	JU394	98	0.00	0.00	3.69
**10**	JU561	110	0.00	0.00	3.30
**11**	MY10	117	0.00	0.00	3.10
**12**	N2	533	0.00	0.00	0.69
**13**	JU363	129	0.78	0.02	4.24
**14**	JU1409	122	0.82	0.02	4.48
**15**	JU1200	119	0.84	0.02	4.59
**16**	JU1242	117	0.85	0.02	4.67
**17**	JU751	117	0.85	0.02	4.67
**18**	JU440	113	0.88	0.02	4.83
**19**	EG4349	109	0.92	0.02	5.01
**20**	MY14	107	0.93	0.02	5.10
**21**	JU1652	99	1.01	0.03	5.50
**22**	PB303	98	1.02	0.03	5.55
**23**	ED3046	94	1.06	0.03	5.79
**24**	JU346	91	1.10	0.03	5.97
**25**	JU830	322	1.55	0.51	3.59
**26**	ED3049	118	1.69	0.21	5.99
**27**	CB4852	113	1.77	0.22	6.25
**28**	JU642	113	1.77	0.22	6.25
**29**	JU1400	112	1.79	0.22	6.30
**30**	JU322	112	1.79	0.22	6.30
**31**	JU1896	111	1.80	0.22	6.36
**32**	JU323	109	1.83	0.22	6.47
**33**	CX11285	108	1.85	0.23	6.53
**34**	DL238	107	1.87	0.23	6.59

© The Authors under the terms of the Creative Commons Attribution License http://creativecommons.org/licenses/by/3.0/, which permits unrestricted use, provided the original author and source are credited.

**35**	ED3073	106	1.89	0.23	6.65
**36**	JU847	103	1.94	0.24	6.84
**37**	JU258	96	2.08	0.25	7.32
**38**	ED3048	143	2.10	0.43	6.01
**39**	JU1580	95	2.11	0.26	7.40
**40**	CB4851	93	2.15	0.26	7.55
**41**	CB4932	129	2.33	0.48	6.65
**42**	ED3005	120	2.50	0.52	7.13
**43**	JU345	319	2.51	1.09	4.88
**44**	JT11398	118	2.54	0.53	7.25
**45**	PS2025	117	2.56	0.53	7.31
**46**	ED3077	194	2.58	0.84	5.91
**47**	LKC34	114	2.63	0.55	7.50
**48**	JU397	113	2.65	0.55	7.56
**49**	ED3017	112	2.68	0.56	7.63
**50**	CX11276	148	2.70	0.74	6.78
**51**	PX174	111	2.70	0.56	7.70
**52**	JU1491	108	2.78	0.58	7.90
**53**	MY18	108	2.78	0.58	7.90
**54**	JU774	107	2.80	0.58	7.98
**55**	LSJ1	105	2.86	0.59	8.12
**56**	DL226	103	2.91	0.60	8.28
**57**	ED3052	326	3.07	1.48	5.57
**58**	ED3011	93	3.23	0.67	9.14
**59**	CX11264	119	3.36	0.92	8.38
**60**	JU1395	115	3.48	0.96	8.67

© The Authors under the terms of the Creative Commons Attribution License http://creativecommons.org/licenses/by/3.0/, which permits unrestricted use, provided the original author and source are credited.

**61**	CX11307	114	3.51	0.96	8.74
**62**	CX11314	114	3.51	0.96	8.74
**63**	ED3040	112	3.57	0.98	8.89
**64**	JU1172	109	3.67	1.01	9.13
**65**	CB4853	108	3.70	1.02	9.21
**66**	JU406	108	3.70	1.02	9.21
**67**	JU775	108	3.70	1.02	9.21
**68**	MY23	104	3.85	1.06	9.56
**69**	RC301	104	3.85	1.06	9.56
**70**	CB4858	146	4.11	1.52	8.73
**71**	PX179	121	4.13	1.36	9.38
**72**	EG4724	109	4.59	1.51	10.38
**73**	EG4725	109	4.59	1.51	10.38
**74**	PB306	107	4.67	1.53	10.57
**75**	EG4946	106	4.72	1.55	10.67
**76**	WN2002	100	5.00	1.64	11.28
**77**	JU1213	134	5.22	2.13	10.47
**78**	QX1211	324	5.25	3.09	8.27
**79**	JU367	95	5.26	1.73	11.86
**80**	JU1212	226	5.31	2.77	9.09
**81**	CB4856	731	5.47	3.94	7.38
**82**	EG4347	108	5.56	2.07	11.70
**83**	QX1233	104	5.77	2.15	12.13
**84**	AB1	172	5.81	2.82	10.43
**85**	JU1568	136	5.88	2.57	11.26
**86**	MY1	136	5.88	2.57	11.26

© The Authors under the terms of the Creative Commons Attribution License http://creativecommons.org/licenses/by/3.0/, which permits unrestricted use, provided the original author and source are credited.

**87**	JU311	115	6.09	2.48	12.14
**88**	CX11262	179	6.15	3.11	10.73
**89**	JU792	112	6.25	2.55	12.45
**90**	JU782	90	6.67	2.49	13.95
**91**	JU1581	119	6.72	2.95	12.82
**92**	CX11271	109	7.34	3.22	13.95
**93**	CB4854	104	7.69	3.38	14.60
**94**	JU1586	104	7.69	3.38	14.60
**95**	JU1246	103	7.77	3.41	14.73
**96**	DL200	127	7.87	3.84	14.00
**97**	JU310	107	8.41	3.92	15.37
**98**	MY16	142	8.45	4.44	14.30
**99**	CX11315	235	8.94	5.62	13.34
**100**	JU778	314	11.15	7.89	15.16

© The Authors under the terms of the Creative Commons Attribution License http://creativecommons.org/licenses/by/3.0/, which permits unrestricted use, provided the original author and source are credited.

Supplementary material for [Alcorn, MR et al], [2016], [Heterotaxy in Caenorhabditis: widespread natural variation in L/R arrangement of the major organs], *Phil. Trans. R. Soc. B*.
doi: 10.1098/rstb.2015.0404

Supplementary Figure 2: Permuted *P* values from Genome-Wide Association Study (GWAS). N = 10,000 permutations. Permuted *p* values for each SNP were calculated using a linear model that associates the genotypes of each strain (N = 100 isolates representing 97 unique haplotypes) with permuted, categorical phenotype. Chromosomes are indicated by the numbered boxes (I-V, X) and genomic position along the chromosome is represented in Megabasepairs (Mb) on the x-axis. $-\log_{10}$ of each *p* value is represented on the y-axis.

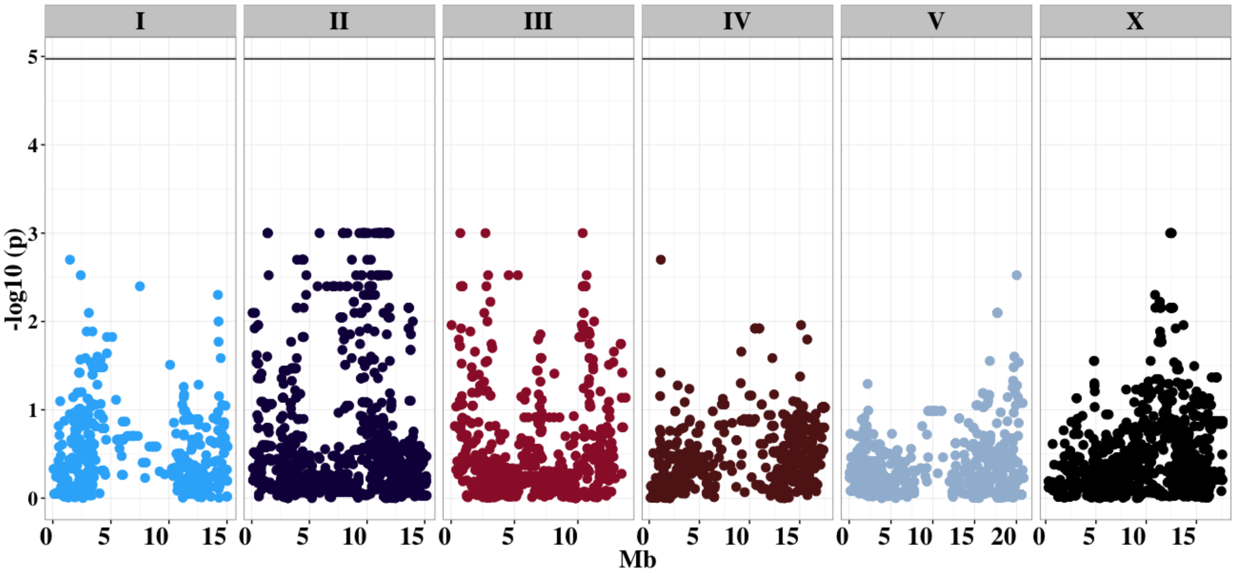

Supplementary Figure 3: Frequency of Heterotaxy in *C. elegans* natural isolates is a temperature-sensitive trait in males. Reversals in males of *C. elegans* isolates cultured at 20˚C and 25˚C. Sample size per isolate ranges from 90 < n < 750 at 20˚C and 40 < n < 700 at 25˚C. Black bars represent 95% Binomial proportion confidence intervals calculated using Clopper-Pearson/Exact method (See Materials and Methods).

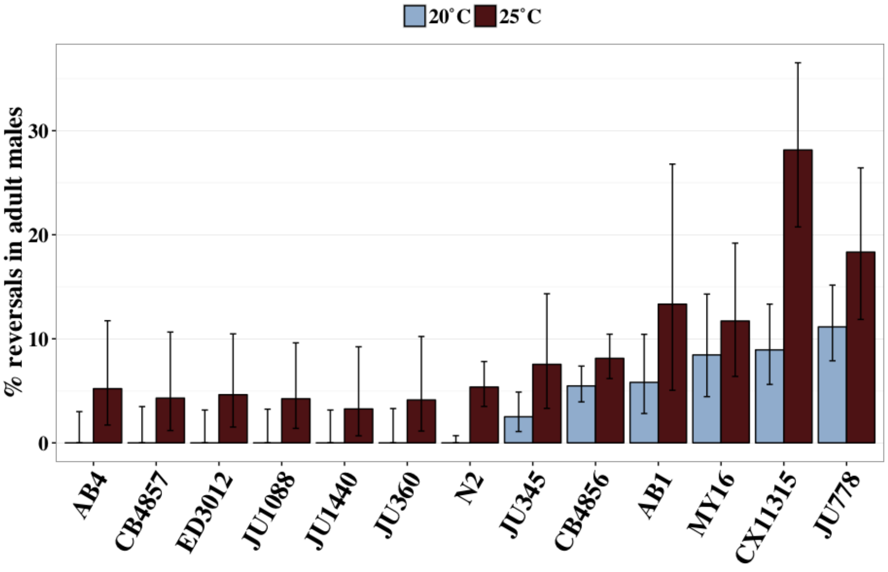

© The Authors under the terms of the Creative Commons Attribution License http://creativecommons.org/licenses/by/3.0/, which permits unrestricted use, provided the original author and source are credited.

Supplementary material for [Alcorn, MR et al], [2016], [Heterotaxy in Caenorhabditis: widespread natural variation in L/R arrangement of the major organs], *Phil. Trans. R. Soc. B.* doi: 10.1098/rstb.2015.0404

Supplemental Figure 4: Complementary data for Figure 7: The propensity for L/R gut-gonad reversals varies widely in adult males of RILs at 20˚C. RILs are in ascending order. Black bars represent lower and upper bounds for 95% Binomial proportion confidence intervals calculated using the Clopper-Pearson/Exact method (See Materials and Methods).

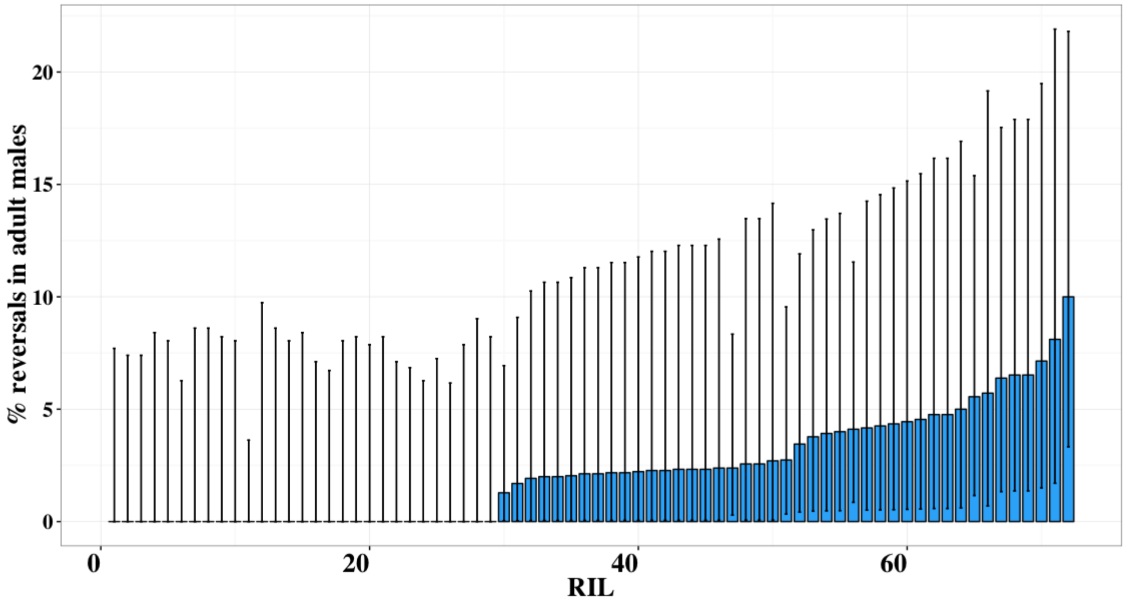

Supplemental Table 2: Complementary data for Figure 7: The propensity for L/R gut-gonad reversals widely in adult males of RILs at 20˚C. This table includes: RIL name, total sample size, percent reversals and the lower and upper bounds for 95% Binomial proportion confidence intervals calculating using the Clopper-Pearson/Exact method (See Materials and Metods).

RIL Number	RIL name	Total Sample Size	Reversals %	Lower Bound %	Upper Bound %
1	JR3703	46	0.00	0	7.71
2	JR3709	48	0.00	0	7.40
3	JR3714	48	0.00	0	7.40
4	JR3716	42	0.00	0	8.41
5	JR3720	44	0.00	0	8.04
6	JR3723	57	0.00	0	6.27
7	JR3725	41	0.00	0	8.60
8	JR3727	41	0.00	0	8.60
9	JR3728	43	0.00	0	8.22
10	JR3729	44	0.00	0	8.04

© The Authors under the terms of the Creative Commons Attribution License http://creativecommons.org/licenses/by/3.0/, which permits unrestricted use, provided the original author and source are credited.

**11**	JR3734	100	0.00	0	3.62
**12**	JR3738	36	0.00	0	9.74
**13**	JR3739	41	0.00	0	8.60
**14**	JR3741	44	0.00	0	8.04
**15**	JR3742	42	0.00	0	8.41
**16**	JR3744	50	0.00	0	7.11
**17**	JR3745	53	0.00	0	6.72
**18**	JR3747	44	0.00	0	8.04
**19**	JR3750	43	0.00	0	8.22
**20**	JR3752	45	0.00	0	7.87
**21**	JR3753	43	0.00	0	8.22
**22**	JR3754	50	0.00	0	7.11
**23**	JR3757	52	0.00	0	6.85
**24**	JR3758	57	0.00	0	6.27
**25**	JR3763	49	0.00	0	7.25
**26**	JR3767	58	0.00	0	6.16
**27**	JR3770	45	0.00	0	7.87
**28**	JR3772	39	0.00	0	9.03
**29**	JR3774	43	0.00	0	8.22
**30**	JR3707	78	1.28	0.03	6.94
**31**	JR3731	59	1.69	0.04	9.09
**32**	JR3756	52	1.92	0.05	10.26
**33**	JR3732	50	2.00	0.05	10.65
**34**	JR3737	50	2.00	0.05	10.65
**35**	JR3748	49	2.04	0.05	10.85
**36**	JR3701	47	2.13	0.05	11.29

© The Authors under the terms of the Creative Commons Attribution License http://creativecommons.org/licenses/by/3.0/, which permits unrestricted use, provided the original author and source are credited.

**37**	JR3711	47	2.13	0.05	11.29
**38**	JR3699	46	2.17	0.06	11.53
**39**	JR3724	46	2.17	0.06	11.53
**40**	JR3733	45	2.22	0.06	11.77
**41**	JR3726	44	2.27	0.06	12.02
**42**	JR3755	44	2.27	0.06	12.02
**43**	JR3708	43	2.33	0.06	12.29
**44**	JR3712	43	2.33	0.06	12.29
**45**	JR3760	43	2.33	0.06	12.29
**46**	JR3740	42	2.38	0.06	12.57
**47**	JR3743	84	2.38	0.29	8.34
**48**	JR3698	39	2.56	0.06	13.48
**49**	JR3751	39	2.56	0.06	13.48
**50**	JR3718	37	2.70	0.07	14.16
**51**	JR3722	73	2.74	0.33	9.55
**52**	JR3762	58	3.45	0.42	11.91
**53**	JR3765	53	3.77	0.46	12.98
**54**	JR3697	51	3.92	0.48	13.46
**55**	JR3736	50	4.00	0.49	13.71
**56**	JR3749	73	4.11	0.86	11.54
**57**	JR3706	48	4.17	0.51	14.25
**58**	JR3693	47	4.26	0.52	14.54
**59**	JR3746	46	4.35	0.53	14.84
**60**	JR3702	45	4.44	0.54	15.15
**61**	JR3766	44	4.55	0.56	15.47
**62**	JR3710	42	4.76	0.58	16.16

© The Authors under the terms of the Creative Commons Attribution License http://creativecommons.org/licenses/by/3.0/, which permits unrestricted use, provided the original author and source are credited.

**63**	JR3735	42	4.76	0.58	16.16
**64**	JR3721	40	5.00	0.61	16.92
**65**	JR3696	54	5.56	1.16	15.39
**66**	JR3713	35	5.71	0.70	19.16
**67**	JR3695	47	6.38	1.34	17.54
**68**	JR3700	46	6.52	1.37	17.90
**69**	JR3719	46	6.52	1.37	17.90
**70**	JR3730	42	7.14	1.50	19.48
**71**	JR3764	37	8.11	1.70	21.91
**72**	JR3715	50	10.00	3.33	21.81

© The Authors under the terms of the Creative Commons Attribution License http://creativecommons.org/licenses/by/3.0/, which permits unrestricted use, provided the original author and source are credited.
